# Supplementary material for: Preferred music-listening level in musicians and non-musicians
Source: PLoS One. 2022 Dec 21;17(12):e0278845. doi: 10.1371/journal.pone.0278845 (PMC9770423; doi:10.1371/journal.pone.0278845)
Supplement: S1 Table — (DOCX) [file pone.0278845.s001.docx]

**S1 Table**. Levels of the music samples in dB(A) when played through the audiometer.

| **Music Sample**  **(and Genre)** | **Audiometer Level Display [dB HL]** | **FFT^a^ [dB(A)]** | **Maximum^b^ [dB(A)]** |
| --- | --- | --- | --- |
| (1) Virtual Insanity:  Jamiroquai (90s) | 70 | 75.9 | 92.3 |
|  | 80 | 85.7 | 101.3 |
|  | 90 | 95.4 | 111.2 |
|  | 100 | 105.2 | 121 |
| (2) Whole Lotta Love:  Led Zeppelin (Classic Rock) | 70 | 76.9 | 93 |
|  | 80 | 86.2 | 102 |
|  | 90 | 95.8 | 111.9 |
|  | 100 | 106 | 122 |
| (3) Crazy in Love:  Beyoncé (Pop) | 70 | 75.5 | 93.8 |
|  | 80 | 85 | 102.3 |
|  | 90 | 95.2 | 111.6 |
|  | 100 | 104.4 | 122.4 |
| (4) Sad but True:  Metallica (Metal) | 70 | 75.4 | 92.9 |
|  | 80 | 85.7 | 103 |
|  | 90 | 94.4 | 110.6 |
|  | 100 | 104.7 | 121.8 |
| (5) Heartbeats:  Jose Gonzalez (Acoustic) | 70 | 76 | 93.2 |
|  | 80 | 85.3 | 102.7 |
|  | 90 | 94.3 | 111 |
|  | 100 | 105.9 | 122 |
| (6) Symphony No. 5 in C Minor:  Ludwig van Beethoven (Classical) | 70 | 75.7 | 93.1 |
|  | 80 | 86 | 102.9 |
|  | 90 | 95.1 | 110.9 |
|  | 100 | 105.4 | 121.4 |
| (7) Environmental  Sounds/Bird Song | 70 | 76.2 | 92.8 |
|  | 80 | 85.8 | 102 |
|  | 90 | 95 | 110.7 |
|  | 100 | 105.7 | 121.9 |
| **Average “Correction”** |  | **5.5** | **22** |

^a^ Fast Fourier transform average dB(A) reading when measured at one octave wide window at 500Hz for duration of 60 seconds.

^b^ Maximum dB(A) reading when measured at one octave wide window at 500 Hz, for duration of 60 seconds.
